# How do Law Students Develop Commercial Awareness? Listening to the Student Voice on the Roles of the Law School and the Law Student in Developing Commercial Awareness

**DOI:** 10.1007/s10991-023-09321-1

**Published:** 2023-01-28

**Authors:** Siobhan McConnell

**Affiliations:** School of Law, Northumbria University, Newcastle, UK

**Keywords:** Commercial awareness, Development, Law school, Employability, Law firms

## Abstract

This article provides an authentic, student-centred account of how law students develop their commercial awareness on their journey to graduate employment. Drawing on data collected from a two year research study involving law students going through the graduate interview process, this article presents the first detailed empirical findings on how, when and why law students develop their commercial awareness. This data is important because law students have a wide range of available career options and commercial awareness is required across a range of graduate professions, including the legal sector. The findings of this study indicate that the law school played a part in developing law students’ commercial awareness but that its role was limited due to a lack of explicit guidance on what commercial awareness meant and how teaching and learning activities supported development. Students identified development much more frequently and explicitly through their own independent developmental activities. As well as providing valuable insight into law student perceptions of the role of the law school and the law student in developing commercial awareness, this article makes recommendations for legal educators on how to support students in developing their commercial awareness.

## Introduction

Commercial awareness is perceived by employers to be an essential graduate skill and it is required across the graduate recruitment spectrum.^1^ Although there is no agreed definition of commercial awareness, generally, it can be said to encompass the ability to understand an employer business and the sector in which it operates.^2^ Surveys of employers find that graduates lack commercial awareness^3^ and there is a perceived skills gap in relation to it.^4^ Whilst such surveys include the views of law firms, where many law students aspire to work, the survey findings are not specific to the legal sector. However, the Legal Education and Training Review (LETR 2013), the most recent major review into legal education and training, noted the significance of commercial awareness to the legal profession.^5^ The findings of the LETR reflected the fact that commercial awareness has become more important to the legal profession over the last 40 years as it continues its transition to a more commercialised setting.^6^ This article will consider how this change in focus began in larger commercial law firms. The move to a more commercial emphasis within those firms has influenced their recruitment practices which, in turn, have impacted on law students who want to work in the legal sector. The larger commercial law firms dominate the legal profession in terms of the recruitment of future solicitors. In 2020–2021 there were 5,495 traineeship registrations.^7^ Almost one third of all traineeship registrations were in the City of London, where many of the biggest commercial law firms are based, and over 50% of traineeships were in larger firms.^8^ Given their position within the recruitment market, these larger firms dictate the skills and attributes required of future lawyers.^9^ The recruitment literature used by such firms highlights the importance of commercial awareness to the legal sector and those seeking access to it.^10^ Despite the importance of commercial awareness to students seeking to join the legal sector and other graduate professions, there is limited empirical evidence of how law students develop commercial awareness, leaving a gap in our understanding of this key graduate skill. This article presents the findings of a two year research study examining law students’ perspectives on how they develop commercial awareness during their time at university. The study provides the first detailed exploration of how, when and why law students seeking graduate employment develop commercial awareness and considers the related implications for legal educators.

This article begins with an overview of commercial awareness and its importance to the legal sector. This article then considers the existing literature on how law students develop commercial awareness before presenting the research method and the findings. This study found that although the law school (through its taught modules and co-curricular activities) played a role in developing law students’ commercial awareness, from a student perspective its impact was fairly limited. Students were much more likely to link their commercial awareness development to their own activities, providing explicit examples of how they sought to enhance their commercial awareness independently. Influenced by key stakeholders in the legal profession, many of the students in this study adopted a strategic, long-term approach to development in order to match perceived employer requirements. The student voice is key in understanding how students regard employability and engage with university support. It is also critical in identifying what students find helpful to move into employment.^11^ There is much that law schools can learn from the student voices heard in this article in terms of how law students develop their commercial awareness and how law schools can support that development.

### What is Commercial Awareness?

The term ‘commercial awareness’ is considered difficult to define and there is evidence that employers and students define it in multiple and differing ways.^12^ A systematic literature review conducted by the author in 2020 examined the existing literature on commercial awareness in the context of the employability of law students in England and Wales.^13^ As part of the review, the analysis considered the range of definitions of commercial awareness found in the literature. The LETR employed a broad definition, based on careers advisers’ (rather than law firm) views, noting it was a ‘composite concept’ (LETR 2013, para. 2.75). That definition included a variety of knowledge, skills and attributes, for example, knowledge of business issues, current affairs and client sectors. It also incorporated an understanding of law as a business, legal practice management and the ability to appreciate a client’s wider commercial objectives rather than focusing on purely legal solutions.^14^ The systematic review found there was limited empirical research on how law firms define commercial awareness. Useful insight came from a study by Strevens et al. (2011) that examined law firm definitions, finding a focus on understanding that a law firm is a business. The authors also found that commercial awareness encompassed understanding the ‘wider picture than the black letter law applied to a given set of facts,’^15^ echoing the aspect of the LETR definition that acknowledged the importance of legal solutions that focused on client objectives rather than the technical application of legal principles. The conceptual literature focused on commercial awareness meaning an understanding of the law firm as a business. Huxley-Binns (2011, p. 304) argued that, for law students to succeed in the legal profession, legal education should include an understanding of business issues such as ‘profit and loss…client relations, strategy, mission.’ Law firm websites and professional journals^16^ provide additional insight into what commercial awareness means, and focus on an understanding of current affairs, business matters and how political, economic, social and technological issues impact on clients and their sectors. As a result of the systematic literature review, the author suggested that, in a legal setting, commercial awareness meant understanding: (1) law firms, their clients and the sectors in which they operate; (2) how external influences (political, social, economic and technological) impact on law firms, clients and their respective sectors and the advice law firms provide; (3) that the legal rights and remedies of clients may not always best suit their objectives; and (4) that a law firm is a business - lawyers need to make money to stay in business.^17^ Given this definition, it is necessary to consider the importance of commercial awareness to law firms and whether it is required at the point of entry to those firms.^18^

## The Legal Sector Context

### The Importance of Commercial Awareness to Commercial Law Firms

The systematic review found that the phrase ‘commercial awareness’ became more prevalent in the 1980s, a time of significant change in the legal profession in terms of regulation, marketing and competition. Client surveys from that period highlighted the importance of commercial awareness to the banking and corporate clients of large London law firms.^19^ In the 1990s, Hanlon and Jackson (1999) found that larger commercial clients demanded commercially aware lawyers or they would take their legal work elsewhere. The importance of commercial awareness to law firms^20^ appears rooted in the ongoing commercialisation of the profession.^21^ As such, commercial awareness has typically been associated with commercial law firms that no longer compete for business solely on the basis of legal expertise. Such firms are now business advisers, working in partnership with clients, offering knowledge and understanding of their clients’ businesses and sectors.^22^ A study of larger corporate law firms by Sommerlad (2011) highlighted the drive within those firms to provide clients with entrepreneurial legal practitioners who offered excellent legal knowledge but also prioritised commerciality. Sommerlad also documented the change in the lawyer-client relationship – lawyers were no longer on a pedestal but were part of a service industry and as such needed to show real understanding of their clients.^23^

Whilst initially commercial awareness was a skill that was associated with in-house lawyers^24^ or needed to acquire promotion and/or partnership, it is now expected by many firms at the start of a legal career. The recruitment literature employed by large commercial law firms demonstrates its importance during the graduate recruitment process.^25^ Further evidence comes from Thomas’s (2018) review of the graduate recruitment webpages of 50 top UK law firms, where it was found that 30 firms mentioned commercial awareness. In interviews with legal professionals from 17 law firms, Thomas found that the majority thought commercial awareness was a highly desirable skill.^26^ Similarly, in interviews with Hong Kong law firms (including UK and US based firms that recruit globally) on the skills needed to gain employment, Mitchard (2022) found that participants from medium to large firms thought candidates lacked commerciality. Candidates needed to ‘transition from seeing law as an academic exercise’ to understanding that real life legal issues may not have clear answers (Mitchard 2022, p. 12). They also needed more knowledge of the business of law i.e. understanding law firm management and maintaining profitability. Together these studies indicate that commercial law firms require commercially aware employees. However, it must be considered whether this requirement applies to other types of law firm.

### Is Commercial Awareness Important to All Law Firms?

The LETR (2013, para. 2.74) recognised the importance of commercial awareness across the profession − 68.9% of respondent practitioners thought knowledge of the business context of law ‘very important or important’ in their work, ranking it ahead of some areas of core legal knowledge. The LETR (2013, para. 2.74) noted that an emphasis on commercial awareness was a ‘recurrent finding’ in its research but that support for its importance was not always consistent.^27^ The definition provided by the LETR suggests that the need for commercially aware lawyers should not be reserved to commercial practice. For example, the definition included understanding current affairs, legal practice management and wider client objectives – an understanding of these factors is critical for working in all legal settings. Further, it is arguable that the commercial awareness narratives of the larger commercial firms may be replicated across the profession as their lawyers move to different firms, specialisms and locations. There is some additional empirical evidence that commercial awareness is relevant to other firm types. Strevens et al. (2011, p. 341) suggested that commercial awareness was a ‘universal employability trait’ required across the sector. Williams et al. (2019, para. 6.3.1 and 7.2) conducted a study involving a range of legal sector employers, finding that commercial awareness was an ‘important quality’ for qualified staff. Nicholson (2022) found that law firms specialising in a variety of practice areas wanted students to understand that law firms are businesses, confirming the importance of this aspect of commercial awareness to the profession.

Overall, there appears to be some compelling evidence that various types of law firm both value and demand commercially aware employees. This is reinforced by a range of law student focused websites (offering careers guidance and employment opportunities) that highlight the importance of commercial awareness to the profession and those seeking access to it.^28^ This study provides insight into the impact of the discourse employed by these stakeholders on students who were applying to a range of firms and a variety of roles within those firms.

### Do Law Firms Assess Commercial Awareness During the Graduate Recruitment Process?

If it is accepted that commercial awareness is an important graduate skill, then it is necessary to consider if law firms actually assess students on their commercial awareness. The focus on commercial awareness in law firm recruitment literature is suggestive that it will be assessed as part of the recruitment process. The academic literature on how and when commercial awareness is assessed during the graduate recruitment process is fairly limited but the existing evidence suggests assessment is commonplace.^29^ Aligning with the previous discussion of the importance of commercial awareness to commercial law firms, Sommerlad (2011) found in her study of corporate law firm recruitment practices that employers assessed commercial awareness in applications, at interviews and at assessment centres. Similarly, Etherington (2016) found all but one of the six law firms of varying types and sizes that he interviewed assessed candidates’ commercial awareness. Of the 28 students he surveyed, 89% stated it had been assessed. Other studies have argued commercial awareness is beneficial for long term employability within the profession.^30^ All of these findings have repercussions for law students seeking to meet employer expectations; they must ‘warrant and perform’ their commercial awareness early in the recruitment process (Tomlinson 2017 p. 345). Naturally, this has implications for universities and their law schools in terms of how to support students in developing their commercial awareness.

## Commercial Awareness, Universities and Law Schools

### Commercial Awareness and the Higher Education Landscape

As part of the neo-liberal agenda, employers have pushed responsibility for skills development on to universities.^31^ Universities are expected to ensure that graduates have the skill sets that employers require – in theory, this saves employers time and money. There is a purported mismatch between the skills employers demand and the skills universities provide.^32^ In effect, universities are under pressure from various stakeholders – employers (who demand employment ready graduates), the government (that expects universities to provide a skilled workforce) and perhaps, most importantly, students who, as consumers of education, anticipate more than the acquisition of a good degree result when paying for their degree. The employability agenda is critical to universities. Employment after graduation is a progression indicator that will be used to rate universities in the revised Teaching Excellence Framework.^33^ It is also an important factor in university league table rankings that influence students in their choice of university.^34^

Studies involving non-law disciplines have highlighted the need for universities to consider commercial awareness more.^35^ For example, Sarkar et al. (2020) found little focus on the development of commercial awareness in science degrees, arguing that this lack of emphasis contrasted with the importance of commercial awareness to employers. This limited focus would disadvantage graduates from an employability perspective because they would exit university with little understanding of the importance of commercial awareness to employers. As will be argued shortly, these findings perhaps show parallels with the position of law students seeking graduate employment. Wilkinson and Aspinall (2007) found that some employers believed universities have a role in developing students’ commercial awareness. However, overall there is limited empirical evidence on how commercial awareness development takes place, what works and what does not and what support students actually want and need.^36^

### Commercial Awareness and the Law School

For law schools, any debate around the importance of employability skills like commercial awareness in an undergraduate degree must be seen in the context of the ‘supply and demand’ side of the legal profession. The significant changes that are taking place in the pathway to qualification as a solicitor must also be considered.

Competition for graduate employment within the profession grows each year. In 2021, 18,927 students graduated from a first law degree in England and Wales, the highest number on record. 81% of those students graduated with a first or upper second class degree.^37^ As previously noted, there were 5,495 training contract vacancies in 2020–2021. For students seeking a career at the Bar, there were 371 available pupillages.^38^ Law graduates also join the profession by becoming paralegals. The number of paralegal vacancies each year is unknown but employing paralegals has become more popular in the legal sector in recent years as employers seek to engage graduates to carry out legal work at a lower salary to that of a trainee and without the commitment of a training contract.^39^ The introduction of the Solicitors Qualifying Examinations (SQE)^40^ as the main pathway to qualification as a solicitor will make working as a paralegal more appealing from a student viewpoint. This is because there will no longer be a ‘training contract barrier’ to qualification. To qualify as a solicitor, graduates will need to pass two centralised examinations and complete two years of qualifying work experience. Qualifying work experience does not need to be in the format of a traditional two year training contract and so paralegal work will constitute qualifying work experience. The landscape for legal employment is even more competitive than it first appears, given that non-law graduates can join the profession by taking additional qualifications, a trend that may well increase as the SQE becomes more established. As such, employers have significant choice in whom they seek to recruit. Law schools have considerable choice, and perhaps challenge, in how they seek to develop their curricula to ensure that their students are placed in the best possible position given the competitive nature of the legal recruitment market.

In terms of guidance for law schools on teaching commercial awareness, the LETR (2013, para. 2.74 and 2.173) recognised the importance of commercial awareness to the profession, identifying a knowledge and skills gap in relation to it. The LETR stated that because commercial awareness was not a priority for all legal service providers, it should not be a formal requirement of the academic stage of legal study but instead a more explicit aspect of the Legal Practice Course (LPC), particularly for students pursuing a career in commercial or corporate law.^41^ The LETR approach seemed at odds with the nature of legal recruitment. Highly competitive and well-paid training contract opportunities, offered by larger law firms, form, as noted, a significant number of available training contracts.^42^ Such roles often require students to have or at least understand commercial awareness^43^ and target second year undergraduate law students. Some law firm graduate recruitment schemes now target first year students.^44^ Omitting commercial awareness from the undergraduate degree potentially excludes students from accessing these opportunities as well as vacancies arising later. Huxley-Binns (2011) predicted (correctly) that many law students would become paralegals without doing the LPC; paralegals also need to be commercially aware. The introduction of the SQE means that including commercial awareness in the undergraduate curriculum will perhaps be even more critical. Unlike the LPC that had a remit to develop students’ commercial awareness, the SQE assessment specifications make no explicit reference to commercial awareness.^45^ The assessments test the Solicitors Regulation Authority Statement of Solicitor Competence that seemingly includes aspects of commercial awareness, such as understanding client objectives and the commercial context in which solicitors work.^46^ However, there is no explicit mention of commercial awareness and, unlike the LPC, no remit to teach it.

The draft Subject Benchmark Statement for Law, which provides guidance on the content and delivery of law degrees, also does not mention commercial awareness.^47^ However, the draft stipulates that the threshold level skills required to be developed within a law degree include employability skills and an awareness of the future of the legal profession (QAA 2022, pp.11–12). These factors were not included in the previous statement (QAA 2019) and the revised draft provides explicit acknowledgement of the need for the development of employability skills within law degrees. Although it will be up to law schools to interpret the finalised statement for their own purposes, a focus on incorporating employability skills into curricula is likely to be required. A balanced approach to curriculum development that satisfies the need for both intellectual capacity and skills in the modern graduate workplace is vital. From a commercial awareness perspective, it is likely that many larger law firms will continue to recruit second year students. This approach, together with the fact that many law students will seek work in law firms on graduation, either through a traditional traineeship or by working as a paralegal, means the need to develop law students’ commercial awareness during their undergraduate degree remains critical. It must also be remembered that many law students will obtain careers outside of the legal profession^48^ and, as noted, commercial awareness is required by employers across a range of graduate roles,^49^ for example, in business, finance, human resources and the public sector.

## Existing Literature on the Development of Commercial Awareness in Law Schools

Collier (2014) reflected on the influence of the neo-liberal agenda in law schools, suggesting that universities were now stakeholders in a global knowledge economy. One aspect of this process was the incorporation by law schools of commercial awareness into curricula. This current study considers student views on whether commercial awareness *is* incorporated into curricula and how effective that inclusion is. There is evidence that some within the profession doubt whether law schools can develop commercial awareness. The employer participants in Strevens et al’s (2011) study thought commercial awareness could not be taught in university and had to be learnt in the workplace. The authors disagreed, stating simulated clinical legal work could develop both practical and academic understanding of the law, for example, the financial implications of a civil claim. Some literature assumes development may occur in various contexts but there is limited empirical research to substantiate this position.^50^

The role of clinical legal education (CLE) and pro bono work in developing commercial awareness alongside other employability skills has been the subject of some useful empirical research.^51^ A small-scale international study by Cantatore et al. (2021) identified that pro bono work improved students’ industry awareness,^52^ providing them with a better understanding of legal practice and how firms operate. In contrast, a study by Alexander and Boothby (2018) found that alumni participants working in the legal profession, who had experienced CLE whilst at university, did not feel that clinic had developed their commercial awareness. The authors noted the challenge in providing an authentic experience of the commercial dynamics of practice in a CLE setting. The viewpoint of law firms on whether CLE could develop commercial awareness indicated that the profession had mixed views (Thomas 2018). Whilst some participants thought CLE could enhance commerciality, others suggested it would have limited impact, with one noting it would not provide students with experience of costing and billing legal work.^53^

The author has conducted empirical research that focuses solely on the development of commercial awareness in CLE in a mixed methods study involving supervisors and students teaching and learning in the CLE facility in her law school.^54^ Students were asked whether clinic had helped to develop their commercial awareness and, if so, how. Supervisors were asked to outline the teaching and learning activities they used to develop students’ commercial awareness. The findings indicated that some students perceived that they developed their commercial awareness in clinic, with some showing more profound development than others. Some students gained a better understanding of what commercial awareness meant. However, the potential for CLE to develop commercial awareness was fairly limited. Supervisors used a range of inventive and realistic activities to enhance students’ commercial awareness but these activities were not always identified by students as developing their commercial awareness. For example, many supervisors were keen to develop student understanding that a law firm is a business, using exercises involving time recording and valuing client work, but there was very limited discussion by students of these activities as examples of how they had developed their commercial awareness. Supervisors named many more commercial awareness enhancement activities than their students. There was very little explicit signposting of the teaching of commercial awareness by the supervisors – many did not link the commercial awareness teaching activity to its development and few supervisors used the term ‘commercial awareness’ when delivering teaching activities.

The above studies provide insight into the role of CLE in developing commercial awareness, however more research is needed on how development takes place across the broader programme of an undergraduate law degree. Overall, there is a lack of robust evidence on how, when and why law students develop commercial awareness. This study aims to address that knowledge gap.

## Method

This article draws on data collected from a research study into a practice interview scheme operating in the law school at Northumbria University, a post-92 university. The scheme provides students with a practice interview prior to a graduate interview. Between February 2019 and July 2021, students requesting a practice interview were invited to participate in the research study. Ethics approval was obtained from the author’s institution prior to starting the data collection process and each participant provided informed consent. Participants completed questionnaires before and after their practice interview and attended a semi-structured interview after their graduate interview to discuss their interview experience. Out of 33 participants, 22 participants returned for the semi-structured interview. Table 1 contains key participant data relating to those participants. The study produced a significant amount of quantitative and qualitative data concerning the role of commercial awareness in the law student journey to graduate employment. This article focuses on the qualitative data produced during the semi-structured interviews concerning how, when and why participants developed their commercial awareness. Most participants (90.90%) applied for roles in law firms, providing useful insight into how they sought to develop their commercial awareness to access legal employment. The experiences of the participants who were applying for graduate roles outside of the legal profession are included because their accounts add to our understanding of how law students develop their commercial awareness whilst at university. Each semi-structured interview lasted 20–40 min, was audio-recorded and professionally transcribed. The transcripts were thematically analysed^55^ using NVivo software.

**Table 1:** Participant Data

Participant	Year of Study	Employer Type	Role Applied For
P1	4	CL	VS
P2	4	HSL	P
P3	4	CL	VS/TC
P4	4	ICL	VS
P5	4	HSL	P
P6	4	HSL	P
P7	LPC	CL	P
P8	4	ICL	P
P9	4	ICL	P
P10	4	FSL	TC
P11	4	FSL	TC
P12	3	OG	GR
P13	3	ICL	VS
P14	4	OG	GR
P15	2	ICL	TC
P16	LPC	CL	TC
P17	3	FSL	TC
P18	2	CL	VS/TC
P19	LPC	FSL	TC
P20	4	CL	P
P21	4	CL	VS
P22	LPC	CL	TC

**KEY: Employer Type:** CL - Commercial Law Firm, FSL - Full Service Law Firm, HSL - High Street Law Firm, ICL - International, Commercial Law Firm, OG - Non-law graduate employer. **Role Applied For:** GR - Graduate Role, P - Paralegal, TC - Training Contract, VS - Vacation Scheme

The number of semi-structured interviews was above the average required to see data saturation.^56^ However, there are limitations to this study. The undergraduate participants were from one institution, a post-92 university in north east England. Three postgraduate participants attended Russell Group universities prior to joining Northumbria University. Whilst their input is useful in providing a comparative snapshot of their experience at Russell Group institutions, it cannot fully represent the experience of students from those universities. It would be useful to conduct further comparative research with pre and post-92 university students. Social background, ethnicity and disability were not addressed in this study and it would be instructive to consider these factors in future research, particularly given the interest in the impact of social and cultural capital on employability^57^ and on diversity in the legal profession more generally.^58^ Despite these limitations, the findings present valuable information for law schools.

The following discussion focuses on the main themes that the data produced in relation to how participants developed their commercial awareness: (1) the role of the law school and (2) the role of student self-development and the associated sub-themes. It must be noted that this study was exploratory in approach and did not seek to assess which aspects of the author’s suggested definition of commercial awareness had been developed. The findings and discussion section considers which aspects may have been developed, either through explicit identification by participants or by inference. For ease of reference, the definition of commercial awareness is provided again here with each part abbreviated:

(1) law firms, their clients and the sectors in which they operate (‘business and sectoral insight’);

(2) how external influences (political, social, economic and technological) impact on law firms, clients and their respective sectors and the advice law firms provide (‘knowledge of current affairs’);

(3) that the legal rights and remedies of clients may not always best suit their objectives (‘the legal solution is not always the right one’); and

(4) that a law firm is a business - lawyers need to make money to stay in business (‘the law firm as a business’).

Participant quotations are labelled ‘P’ followed by the participant number.

## Findings and Discussion

### The Role of the Law School in Developing Commercial Awareness

#### The Impact of Taught Modules…‘I Think What University is Good at, it Sort of Kicks you into Gear’^59^

There was limited evidence (three participants) that a compulsory first year employability module taken by most of the participants (86.36%) assisted in introducing students to commercial awareness. This module includes a lecture on commercial awareness (what it is, why it is important and guidance on how to develop it) and workshop exercises aimed at enhancing students’ commercial awareness, for example, preparing poster presentations on topical challenges affecting the legal sector. Such modules provide students with their ‘first encounter’ with the employability agenda however many students do not engage with such modules.^60^ Few participants cited the module as a point of development, perhaps because it was so early in their university lives. The majority of participants who had taken this module were in their final year of study and perhaps had forgotten about the module, failed to appreciate its significance or did not attend teaching sessions.

Several participants discussed how taught modules enhanced their commercial awareness by deepening their sectoral knowledge and their understanding of how current affairs, for example political and social issues, impact on the law. Business-focused modules (taken in third or fourth year of undergraduate study or in postgraduate study) were identified by seven participants as sites of development. Participants observed lecturers integrating commercial awareness into their teaching, one stated:

*When we have lectures, they try and relate them back to stuff in the news, so for example in Business Law, we did bankruptcy and they related it back to all the famous bankrupts…they had newspaper articles of pretty much what’s going on in the world…which I think’s developed that because it’s built in with what I’m learning so it’s easier for me to understand the theory, if I’m seeing it in practice. (P11)*

Four participants associated development with early degree non-business modules, such as public law and land law, again linking development to gaining understanding of current affairs. Overall, from the business and non-business modules noted, only two participants stated lecturers were explicit in explaining that the teaching methods employed were designed to develop commercial awareness.

In contrast, four participants recognised explicit development in an optional third year commercial contracts module taught by former solicitors who had practised in commercial law firms. Tutors explained what commercial awareness meant, allocating marks for its demonstration in the module assessment, potentially acting as a spur for development.^61^ When discussing this module, participants identified a focus on ‘the legal solution is not always the right one’ aspect of commercial awareness:

*He really drilled into us the solution is not always legal…we’d be sat in seminars where he’d kind of ask us what the answer was and we’d give it to him and he’d be, like, ‘OK, right, so, you know, you’ve advised the client on what they have to do next based on their legal right but is that’s what’s best for the company?’ and we were all a bit stumped because we’d never really been asked that before, to really think about that kind of effect. (P10)*

Some participants identified commercial awareness was assessed in other modules but assessment was not explicit and nor was the explanation of commercial awareness:

*No I wouldn’t say it was mentioned in the module…it wouldn’t even be implicit in the marks scheme…I only knew because I went to a lecture...(P8)*

It appears that where taught modules were identified as supporting development of commercial awareness, it could be linked to several parts of the suggested definition, i.e. ‘knowledge of current affairs,’ ‘business and sectoral insight’ and, to a lesser extent, ‘the legal solution is not always the right one.’ The data suggests that taught modules can aid development but this seemed to be module and tutor dependent. Most participants had to make the connection between teaching delivery and commercial awareness development for themselves. Participants appeared to develop a deeper understanding in modules where academics explained what commercial awareness was and how the module supported development. In real estate education, a discipline where a significant amount of research around the development of commercial awareness has been conducted, Poon found that when developing commercial awareness during teaching, clear signposting is needed.^62^ The findings here indicate that academics must not assume that students understand commercial awareness and must be clear on what it means in the context of a module and, if relevant, the assessment. As Knight et al. (2003) argue, ‘making the tacit explicit’ is critical. The findings here add to the discourse on the need for universities to ensure that graduates gain, and then are able to present, their higher education experience (educational and otherwise) as ‘positive signals which are likely to be attractive to potential employers’ (Tomlinson 2021, p. 139). By providing clear guidance on what the skills gained are and how and when skills development takes place, universities will place students in a better position from which to articulate their employability to prospective employers during the graduate recruitment process. This will be of ever increasing importance as the number of law graduates increases and competition for employment across the graduate recruitment spectrum grows.

#### The Unrealised Potential of CLE in Developing Commercial Awareness

Fifteen participants had CLE experience at the author’s institution.^63^ There were mixed views on whether their clinic experience assisted in developing commercial awareness, reflecting the current literature.^64^ Working in a business-advice clinic was highlighted as promoting development by three participants, for example, in understanding business start-up issues. This suggested that participants were perhaps developing commercial awareness in terms of enhancing their ‘business and sectoral insight.’ However, identification of how development took place appeared limited:

*I would say SLO*^65^*has in the fact that I worked with a client who had a business…just thinking about sort of learning the set-up you should have for a business and what safeguards you should put in place for a business. I can’t really think of any other examples. (P20)*

Another business-advice participant saw no development during their time in clinic whilst another provided a vague account. This participant focused on supervisor guidance on the importance of commercial awareness but suggested that there was little actual skill development during their time in clinic:

*Not so much with, the clients I’ve got…we haven’t really looked at that…but in some of the firm meetings, we have gone through commercial [awareness]…we’ve just briefly said that in the world today, it is a massive, massive aspect. (P12)*

Two participants who had worked in criminal and employment law clinics saw no developmental role.

Despite suggestions that CLE can support development of a broad understanding of litigation,^66^ only one participant, who had provided housing advice, highlighted its role in developing understanding the ‘legal solution is not always the right one’ aspect of the definition, noting:

*I think that was [X’s], my supervisor, was his biggest thing, he always said ‘It’s great that you always want the best for your client, that’s great, you’ve done all the legal research, clearly they’ve got a claim, but, are they really going to be thanking you in six months when they’re doing their exams and they’ve got court proceedings, potentially to get £300 between four of them?’ (P10)*

None of the participants identified clinic as developing understanding that a law firm is a business and needs to make money – either by explicitly discussing that or through mentioning any activities that link to this aspect of the definition of commercial awareness. The data appears to align with Alexander and Boothby’s (2018) finding that CLE must enable students to experience the commercial realities of practice more but contrasts with other viewpoints that argue CLE provides this insight.^67^ CLE arguably provides an authentic opportunity to provide practical understanding that a law firm is a business, particularly because in the author’s clinic students work in firms advising clients and recording their time. The findings here reflect the author’s conclusions from her empirical study into whether and how CLE develops commercial awareness - there, very few participants identified that their understanding of the ‘law firm as a business’ was developed during their time in clinic.^68^ It appears that there is work to be done in emphasising this aspect of the clinic experience.

As with the other taught modules, there was limited evidence of explicit signposting of commercial awareness development by clinic supervisors. The term was mentioned as a generic skill but rarely flagged in the context of client work. Similarly, Dunn (2017) found that clinic made students aware of the importance of commercial awareness. Dunn concluded that CLE could not replicate commercial awareness in the same way as practice and it was unlikely to be embedded by clinical experience.^69^ This study found limited evidence that CLE enhanced commercial awareness; most participants who had experienced CLE either struggled to connect their clinical experience to their development of commercial awareness or did not mention CLE as assisting with development at all. However there were some encouraging signs that CLE could be used to support a more sophisticated understanding of commercial awareness, suggesting there is potential for CLE to play a greater role in development. Again, the findings here support the results of the author’s separate study, confirming that clearer signposting of CLE teaching activities that support the development of commercial awareness is needed for students to fully benefit from their supervisor’s practice experience and the range of authentic developmental activities on offer.^70^

#### The Importance and Influence of Co-curricular Support

Twelve participants stated that co-curricular activities developed their commercial awareness: networking events, guest lectures and other university arranged employer activities helped, particularly in understanding employer requirements. The co-curricular support referenced by the participants did not link to any particular aspect of commercial awareness but was more aligned with gaining a general knowledge and understanding of commercial awareness, its importance to employers and how it was required when accessing graduate employment. The results echo findings from O’Leary (2017) of the benefits of employability support within degree programmes, i.e. in providing students with a better understanding of employer needs. Similarly, Jorre de St Jorre and Oliver (2018) found employer input valuable and motivating for students.

Six participants appreciated the input of Aspiring Solicitors^71^, an organisation that supports students from underrepresented backgrounds into the legal profession, with one participant noting:

*Aspiring Solicitors, that was a big thing…I joined in the second year and they’re really big on commercial awareness, you’ve got to know it, and I think up until that point I didn’t really know how much…but it’s actually so important in law firms, especially corporate law firms, they really look for it. (P9)*

There is a growing number of similar organisations^72^ providing more opportunities and assistance for students. Like the websites that focus on accessing the legal profession, for example LawCareers.Net, these organisations appear to have a role in reinforcing the rhetoric employed by the larger law firms, ensuring that the need for commercial awareness across the profession becomes more established from a student perspective.

Co-curricular support was clearly influential for the participants in this study. Students now expect co-curricular employability support but whether they engage with it is another matter. The increasing importance of the employability agenda to law schools^73^ and universities in general makes such support critical.

#### A Russell Group Difference?

Only three participants attended Russell Group universities prior to joining Northumbria University so, although this data provides insight into their experience of developing commercial awareness at those institutions, caution should be adopted in determining if there is a Russell Group difference. Further, it must be noted that all three were students on the LPC which places an explicit focus on commercial awareness.^74^ All of these participants stated that their former universities paid little attention to commercial awareness and there was a different approach at Northumbria University, for example:

*But I don’t think the uni [*Russell Group*], in general were that…helpful with that* (commercial awareness), *they didn’t have that many talks or anything. (P18)*

And

*I think module leaders…particularly at Northumbria…focus on developing your commercial awareness as well. I would definitely say that there was more focus at Northumbria. (P22)*

One interpretation of this data could be that, like the participants from the author’s institution who did not engage with an employability module that focused on commercial awareness, these participants simply did not engage in commercial awareness development opportunities provided by their former universities. Another interpretation could be that this data aligns with views that there tends to be less of an employability focus at universities with higher reputational capital (Boden and Nedeva, 2010).^75^ Elite universities have traditionally resisted adopting embedded employability narratives, relying instead on their reputations and the social capital of their students to underpin student success in obtaining graduate employment.^76^ However, it would be unwise to adopt such an interpretation in this study, not only because of the small number of participants that were from Russell Group universities, but because it is clear that the employability agenda is becoming much more important across the university spectrum.^77^ For example, Oxford University has a clear employability offering that focuses on business awareness (a term used interchangeably with commercial awareness).^78^ Wong et al. (2022, p. 1350) recently presented the first study that mapped graduate attributes, the ‘visions that universities aspire of their students, irrespective of their degree discipline.’ They examined the websites of the 76 UK universities with webpages that advertised graduate attributes. 75% of those universities included ‘employability’ in their list of graduate attributes and the authors noted that skills like commercial awareness were mentioned. A study by Nicholson (2021) of law school online prospectuses noted the focus on employability within law schools and it is likely that this will increase in the future, particularly given the importance of the employability agenda to prospective students. Although it is clear that employability narratives are now much more embedded within law schools of all types, it is less clear how skills like commercial awareness are being developed. Further research on how commercial awareness is developed in pre-92 institutions would be beneficial.

### The Role Of Student Self-Development in Developing Commercial Awareness

Although there was some compelling evidence that the degree programme assisted in developing commercial awareness, participants more frequently cited development through participant-led activities. Participants also described more specific examples of how they had independently developed their commercial awareness and appeared confident in those descriptions. As Table 1 demonstrates, participants applied to a variety of types of firm and vacancies. All of the participants tried to develop their commercial awareness in some way during their law degree. The following discussion provides insight into what students did, when they did it and why they decided to focus on commercial awareness when preparing for a graduate interview.

#### What do Students do? The Match and Mismatch Between Activities and Development

Although participants mentioned work experience, part-time work, volunteering, mentoring and networking as assisting with developing commercial awareness, none of these factors stood out in the data analysis as places of development. This is particularly noteworthy in relation to part-time work as this is a source of potential development that could be used to evidence ability. In a study of alumni views of skills development whilst at university, Clark et al. (2016) found that whilst degree and extra-curricular activities developed commercial awareness to the same extent, paid employment developed it significantly. All of the participants in the current study had worked part-time but only one participant acknowledged its role in developing commercial awareness. This lack of focus on the role of part-time work in supporting the development of commercial awareness aligns with Francis’s (2015) findings from a study of law firm recruitment practices. Francis found that employers were receptive to listening to the student ‘story’ on how part-time work in retail could be used to demonstrate commercial awareness but this was not what they were expecting. Francis contended that many students from non-traditional backgrounds, i.e. first generation students from post-92 institutions, would not know that they *can* tell that story or *how* to do so.^79^ There is evidence for this approach in this current study where some participants stated that they thought they should not mention their part-time work to employers at interview and would not know how to use it to demonstrate commercial awareness. One participant stated that she thought she should not tell prospective employers about her work at McDonald’s because she thought it had ‘bad connotations’ and would not impress interviewers. Another felt that their work at Morrisons made them ‘less unique’ and so was reluctant to discuss it. These participants, like many others, needed guidance on how their part-time work could be used to demonstrate commercial awareness, for example, through showing an understanding of their employer business, and how activities like achieving customer satisfaction and meeting sales targets enhanced their commercial awareness.

#### Approaches to Development and the Commercial Awareness ‘habit’

What was much clearer from the participants’ accounts was the self-motivated ‘industry’ of commercial awareness development, where participants actively sought to improve their abilities in understanding and developing various aspects of commercial awareness. Whilst some participants took a basic developmental approach, for example, researching current affairs just before an interview, others employed a more planned, processual approach, adopting a long-term strategy by developing their awareness ‘over time and in interaction with others’ (Holmes 2013 p. 548). Many participants took practical steps to develop an employability narrative that matched employer requirements.

For most participants, individual research was key, and they explained how they utilised websites, newspapers, magazines and podcasts to develop their commercial awareness. Four participants received emailed weekly commercial awareness updates from websites like LawCareers.Net and Lawyer2Be – as noted such websites promote the importance of commercial awareness to the legal profession.^80^ The activities noted would be critical in developing key aspects of commercial awareness, in particular, ‘business and sectoral insight’ and ‘knowledge of current affairs.’ The respondent legal employers in Mitchard’s study emphasised that students should be encouraged to read newspapers and be up to date with news stories and political and economic issues that impacted on businesses.^81^ In this current study prior research was used at interview to evidence commercial awareness, for example:

*For instance, my answer to [*commercial law firm*], that came from an article that I read in The Guardian. (P16)*

Four participants stated that LinkedIn provided insight into the business world, the legal profession and how news stories affect clients:

*LinkedIn’s my new best friend…I love the news, but I was aware…I need some more commercial legal skills. Like I can recite news and that’s all well and good, but if I don’t know what’s happening here and now, it’s not that impressive. So every day I’ll have a quick scroll through LinkedIn…I’m following some really good solicitors. (P17)*

Some participants provided practical details on their developmental approach. One participant worked as the commercial awareness editor for a student journal, drafting articles on current affairs. Another participant explained:

*Over the summer, I was reading things and then also creating summary documents and just keeping notes and, literally, a few bullet points a week, just if they did get any questions in applications and things like that, then I would have been able to look at my documents and just…answer them better. (P20)*

Several participants described their development of commercial awareness as a long-term project that was part of their everyday lives. This ‘habit’ rewarded them with a sophisticated level of commercial awareness, for example:

*I’ve got a regular habit of just reading the business sections of newspapers and just keep an interest in current affairs. And that’s just a habit that I’ve had for a number of years...(P16)*

And

*…if I ever find myself just sat thinking, ‘I’ve nothing to do,’ I think, ‘well, I’ll have a quick flick through LinkedIn’…obviously, the more you know and the more knowledge you have…on random legal issues, you are building your commercial awareness and while you don’t see it at the time, you know, it all adds up. (P17)*

Three participants appeared to be aiming for a more sophisticated level of commercial awareness, evidenced where they considered their ability to predict how business issues would affect law firms and clients, for example:

*I actually started to practise doing it…reading news articles…making notes on them, and I’ve been doing that for quite a while now…I just sort of know general trends rather than…facts about everything, but I know general trends, I can sort of explain what I think’s gonna happen broadly...(P21)*

This approach gave them more confidence in their interviewing abilities, the same participant noting:

*I realised when I could make my interviews a lot better instead of just in the lead-up to the interview preparing and looking at sort of some of the deals that the firm has done and…a few current trends. I realised that if I did it over a period of time, you can just spot trends better and you’re just going to be more comfortable discussing certain issues. (P21)*

Some participants preferred to be self-reliant rather than depending on university for support, for example:

*I think it’s kind of the sort of thing that it’s better for me to self-teach. I think if someone stood and told me things that are commercial awareness, it wouldn’t make sense; it’s more having your own awareness about it. (P18)*

The above discussion indicates that many participants perceived that they developed their commercial awareness through their own individual research activities. This drive for knowledge and understanding may be rooted in their engagement with the employability narratives of law firms, the law school and third party organisations that emphasise the importance of commercial awareness at the point of access to the legal profession.

#### Seeking Differentiation Outside the Degree

The preference for self-reliance led to some participants adopting additional self-motivated learning activities that they identified as being critical to their commercial awareness development. One participant stated that her desire to learn about business stemmed from feeling in competition with students in other disciplines. She explained:

*I’m teaching myself business stuff. I’ve never known it before. There’ll be a lot of people who know a lot about it or do a combined degree with Business, who’ll*

*understand it really, really well and so being able to match up to that is something that I have to work on a lot. (P18)*

Another participant started a Level 2 business administration course during the first COVID-19 lockdown to improve her commercial awareness. These students were seeking differentiation from outside of the law school and its traditional law-focused modules in order to gain a better understanding of business concepts.

Two participants reflected on the role of family businesses in developing their commercial awareness – a factor not observed in any previous discussion of commercial awareness – but the link to it as a site of development was only recent or unexplored, for example:

*…it’s just…conversations that I have with [*family*]. I don’t view them as commercial awareness lessons, it’s just…talking about business. (P14)*

Overall, these participants seemed to perceive that understanding business concepts was a way of giving them an ‘edge’ in the competitive legal recruitment sector. Alongside the ‘edge’ factor provided by understanding more about business, these participants perceived there was a link between understanding business concepts and developing commercial awareness. These views on the importance of business knowledge are reflected in the employer viewpoints noted in some empirical studies. Nicholson (2022) noted the importance of business knowledge and understanding to law firms whilst the respondents in Mitchard’s (2022) study wanted candidates to understand business structures and legal practice management. The importance of business knowledge to students and employers leads to a consideration of how a law degree could be adapted to incorporate insight into business concepts. Bowley (2020) examined a business case study embedded in a corporate law module in an Australian university, assessing whether it improved student understanding of business concepts and terminology. Prior to taking the module, law students with no previous experience of working in or studying business found understanding business concepts and terminology daunting. The results indicated that the module helped law student understanding by placing business concepts into a practical context. If there is potential student demand and need for more knowledge of business concepts, then law schools like the author’s should consider incorporating more business focused content into modules or enabling students to select business options as part of a more wide-ranging law degree. The advent of the SQE may provide law schools with more flexibility in designing law degrees that allow students more choice and the ability to take a range of law and potentially non-law options that suit both their interests and career aspirations.^82^

### When do Law Students Develop their Commercial Awareness?

Most of the participants were in their later years of study with 81.81% in their final year. This provided a valuable and reflective ‘endpoint’ perspective of when these participants had started to develop their commercial awareness. All of the participants stated they had no knowledge or understanding of commercial awareness before joining university but all described improvement during their time at university. Although many participants became aware of the importance of commercial awareness in first or second year, they stated concerted development only began later, a final year participant noting:

*No, I had no idea about it. I didn’t really have any idea about it until, probably, a year ago or something…I didn’t take it that seriously and I didn’t really know anything about it, to be honest. (P21)*

This approach may have led to participants unwittingly excluding themselves from the high number of training contract opportunities, providing job security and financial support,^83^ targeted at second year law students by firms seeking to secure the ‘best’ candidates.^84^ This is of particular concern in post-92 institutions that have traditionally attracted students from lower socio-economic backgrounds and where students already have more challenges in securing employment in such firms.^85^ The position of law students that want to gain graduate employment further on in their degree or just after graduation (either inside or outside the legal profession) must also be considered. They too need to develop their commercial awareness given its importance to employers. For those students, leaving development until their final year may lead to a superficial and underdeveloped understanding of commercial awareness and its importance to graduate employers. This may create difficulties for them during the graduate recruitment process.

### Why do Law Students Develop their Commercial Awareness?

Employer influence was evident in the participant stories of why they chose to become commercially aware, for example:

*I think the law firms are very explicit in saying that that’s what they’re looking for. (P22)*

And

*I think that’s something that I’ve picked up from extra-curricular stuff, like a lot of the virtual vacation schemes and stuff I’ve done over the summer have had lots of commercial awareness focus things. (P15)*

As noted, participants applied to a range of firm types and roles. All of the participants discussed how they had tried to improve their commercial awareness in some way, perhaps providing further confirmation of its perceived importance in obtaining graduate employment. Four participants talked of commercial awareness giving them an ‘edge’ over other candidates. One participant, keen to become a lawyer, reflected on why she became commercially aware, stating:

*Because I really wanted a training contract, so I knew I had to pull my finger out and actually start trying to understand it.* (P21)

Participants noted commercial awareness was mentioned in recruitment literature, at open days and throughout the graduate recruitment process. Handley (2018, p. 250) argued that final year students are ‘particularly susceptible to employer representations’ of what makes graduates employable. For law students, this directing narrative starts earlier, even if they choose to ignore it, and directly influences how they go about making themselves employable. The findings show parallels with research by Gebreiter (2020) involving students seeking employment with elite accountancy firms. He applied Foucault’s work on technologies of power and the self. Employer recruitment practices were part of a disciplinary process that constructed the ‘ideal recruit’ conveyed to students by firms, universities and other organisations. Gebreiter acknowledged this could only be a partial framework for constructing ideal candidates. What was more relevant was the process students underwent to transform into idealised recruits by engaging with employers and their recruitment narratives to understand their requirements, then taking steps to close CV gaps. As with some of this study’s participants, this was a ‘profound and elaborate process’ taking place over time, going beyond standard interview preparation (Gebreiter 2020 p. 245).

## Conclusion

This study provides the first detailed empirical evidence of how, when and why law students develop commercial awareness during their time at university. Whilst the law school programme helped some participants in developing their commercial awareness, the participants’ self-development activities appeared more critical and effective. The approaches to development employed by many participants seem to reflect the commercial awareness rhetoric pushed by the legal profession (faithfully replicated by law schools and other stakeholders) as it continues to become more commercialised. This research arguably provides further evidence of the anticipatory socialisation effects of this rhetoric and the strategic approaches some students adopt to become commercially aware. When developing commercial awareness, these strategists engaged in activities to develop an employability narrative that matched employer requirements.^86^ This state of sophistication does not come naturally to students. The findings here suggest that for these students the law school had a limited role in development – they achieved enhanced commercial awareness through an ongoing, active process of self-development that was very much student-led but heavily influenced by key graduate recruitment stakeholders. The implications and opportunities these findings present for law schools are now considered.

University can and should be a catalyst for developing students’ commercial awareness. Law schools have a responsibility, and a vested interest,^87^ in supporting student development because of the importance of commercial awareness to employers and because of the highly competitive fields into which law students will graduate. Encouraging self-development assists, particularly in highlighting the benefits of the commercial awareness ‘habit.’ Although evidence of the effectiveness of student self-development is compelling, supporting views that commercial awareness can be ‘practised and learned,’ (Black and Turner 2016 p. 63) law schools cannot devolve responsibility to students. Whilst many participants were self-reliant in their development, others were not. Several participants admitted to only focusing on development in their final year. This ‘just in time’ approach must be avoided because of the recruitment methods of law firms that recruit candidates to lucrative training contract opportunities two years in advance. A lack of commercial awareness is particularly concerning for post-92 universities like the author’s that have a high number of students from lower socio-economic backgrounds and where elite firms have traditionally tended not to focus recruitment efforts.^88^ The strategy of those firms is changing as they seek to diversify socially and recruit in ways that more accurately reflect their clients’ workforces and diversity goals whilst embracing the Levelling Up agenda.^89^ When these firms do target post-92 law schools, students must be prepared and not found deficient in their commercial awareness.

Law schools must incorporate commercial awareness into curricula, working in partnership with careers services. This integration into the undergraduate degree programme is particularly important given the current changes in the legal education landscape as LPC programmes close and the SQE starts to take precedence as the main route to qualification. Early intervention through integrated employability modules or careers sessions assists by introducing students to what commercial awareness means and its importance to employers. This stage could emphasise the different facets of commercial awareness and ensure student understanding reflects employer views whilst emphasising the role of part-time work in supporting development. However, as the findings suggest, some students may not engage. A broader strategy is needed and the findings suggest taught modules aid development. This study indicates that commercial awareness is already taught when academics relate the law to current affairs or discuss issues affecting clients, developing key aspects of commercial awareness. However, academics must go further by explaining what commercial awareness is, linking development explicitly to teaching and learning activities, ensuring students make that connection. As Wong et al. (2022, p. 1351) argue, where academics ‘unpack and make clear’ how their degrees contribute to graduate attributes like commercial awareness, they enable students to ‘recognise the transferable qualities of their study’ which assists them as they seek entry to the modern professional workplace. The findings suggest that embedding commercial awareness in assessment supports development but this must be explicit and academics should emphasise the aspects of commercial awareness that they are seeking to assess, for example, knowledge of current affairs. There is a clear, but not yet fully realised, potential for the development of commercial awareness in CLE. This must focus on signposting how student activities enhance commercial awareness, particularly in understanding that a law firm is a business and that the legal solution is not always the best one for a client.

There are of course challenges for law schools – finding the time, space and desire for further skills development is difficult, particularly with the impact of the SQE. Some modules that aid development, particularly CLE, may fall later in degree programmes.^90^ Academics may themselves not understand commercial awareness and may find integrating employability skills into teaching challenging.^91^ Approaches to integration may vary and may depend on academic position, level of experience and confidence in teaching employability skills, particularly more complex skills like commercial awareness. Appropriate support should be provided.^92^ There is clear opportunity here for law schools to bolster their employability offerings, working with students to develop their commercial awareness and adding value to their degrees.^93^ There are a variety of ways in which commercial awareness can be developed and law schools should consider where and when development can be incorporated into curricula. It is hoped that the student voices heard in this article - their authentic accounts of how they developed their commercial awareness - will assist by providing insight and ideas to academics and, in turn, their students.

## Footnotes

1 Institute of Student Employers (ISE) Surveys (2020, 2022) and Sewell and Dacre Pool (2010).

2 Target Jobs (2022).

3 ISE Survey (2020, p. 18), ISE Survey (2021, p. 23) and ISE Survey (2020, p. 25).

4 ISE Survey (2020, p. 20).

5 LETR (2013, para. 2.74).

6 Hanlon (1999), Strevens et al. (2011) and Sommerlad (2011).

7 A 2.3% decrease on the previous year. The Law Society (2022, p. 44).

8 The Law Society (2022, p. 44). 38.5% of traineeships were located in firms with 81 or more partners and a further 13.2% in firms with between 26 and 80 partners, The Law Society (2022, p. 46).

9 Francis (2015, pp. 182–185).

10 Law firm websites (2022).

11 Parutis and Howson (2020).

12 Wilkinson and Aspinall (2007).

13 McConnell (2022a).

14 LETR (2013, para. 2.75).

15 Strevens et al. (2011, p. 341).

16 Law firm websites (2022) and Smyth (2010).

17 McConnell (2022a, pp. 158-9).

18 This study did not seek to assess whether participants developed the aspects of commercial awareness noted here although the findings do consider which aspects could be said to have been developed.

19 Brown and McGirk (1982) and Blackhurst and Stokes (1985).

20 Thomas (2018, p. 141).

21 Hanlon (1999) and Hanlon (1997).

22 Hanlon (1999). See for example https://www.simmons-simmons.com.

23 Sommerlad (2011, p. 86).

24 Bahl (1989).

25 Law firm websites (2022). See also Hogan Lovells https://graduates.hoganlovells.com/opportunities/vacation-scheme showing commercial awareness as one of the ‘key traits’ of a Hogan Lovells lawyer.

26 Thomas (2018, p. 141).

27 Noting opposition from some members of the Bar and lawyers working in the public sector and with ‘personal plight’ clients.

28 See for example the LawCareers.Net commercial awareness hub at https://www.lawcareers.net/commercial-awareness and The Lawyer/Lawyer2Be https://www.thelawyer.com/commercial-awareness.

29 The author has carried out (as yet unpublished) research at her own institution that examines how and when commercial awareness is assessed during graduate interviews. The findings indicate that across a range of firms of varying sizes interviewing for various legal roles, commercial awareness is more likely than not to be assessed at interview.

30 Bentley and Squelch (2014).

31 Tomlinson (2021, p. 135) and Morley (2007).

32 Tomlinson (2021).

33 Teaching Excellence Framework (2022). One of the student outcomes indicators in the Teaching Excellence Framework used to rate universities is graduate progression based on Graduate Outcomes survey data on graduates in employment/further study fifteen months after graduation.

34 For example, the Complete University Guide (2022).

35 Jackson and Chapman (2012, p. 108) and Sarkar et al. (2020, p. 356).

36 Only in real estate education, see Poon and Brownlow (2014).

37 The Law Society (2022, p. 41).

38 Bar Standards Board (2022). 371 individuals starting their first six months of pupillage in 2020/21.

39 Research suggests the number of paralegals and other legal associate professionals is increasing by 1% each year. It is predicted that the number of legal associate professionals will increase by 2% per year and that 2800 will be needed per year – see Williams et al. (2019, paras 7.1 and 7.3).

40 For further information, see https://sqe.sra.org.uk/about-sqe/what-is-the-sqe.

41 LETR (2013, para 4.69 and recommendation 12 p. 289).

42 The Law Society (2022, pp. 42–46).

43 Law firm websites (2022).

44 See for example the first year ‘fast track’ scheme with Weil https://ukrecruiting.weil.com/fast-track-scheme that places an emphasis on developing candidates’ commercial awareness.

45 For SQE assessment specifications see https://sqe.sra.org.uk/exam-arrangements/assessment-information.

46 See https://www.sra.org.uk/solicitors/resources/continuing-competence/cpd/competence-statement/ at A4b, B3a and D3a accessed December 2022.

47 QAA (2022).

48 Williams et al. (2019, para 2.4) noting that between 2011 and 2016 around 35% of law graduates joined the legal profession each year – the proportion had increased to 39% in 2015/16.

49 ISE Surveys (2020, 2021 and 2022).

50 Bleasdale-Hill and Wragg (2013) in clinic, Newbery-Jones (2016) in a module using video resources to develop understanding of the relationship between law and current affairs.

51 Cantatore (2018) and Thanaraj (2017).

52 In the literature, ‘industry awareness’ and ‘business awareness’ are used interchangeably with commercial awareness.

53 Thomas (2018, pp. 142–143).

54 McConnell (2022b).

55 Braun and Clarke (2006).

56 Guest et al. (2006).

57 Ashley and Empson (2013).

58 The Law Society (2020).

59 P3.

60 Parutis and Howson (2020, p. 385).

61 Jorre de St Jorre and Oliver (2018, p. 55).

62 Poon (2014, p. 62) finding most academics explicitly mentioned ‘commercial awareness’ when teaching it, explaining its meaning.

63 Through a year long, credit bearing module.

64 Alexander and Boothby (2018), Cantatore et al. (2021), Thomas (2018) and McConnell (2022b).

65 SLO denotes the Student Law Office, the clinical legal education facility at Northumbria University Law School.

66 Bleasdale-Hill and Wragg (2013).

67 Marson et al. (2005), Cantatore et al. (2021) and Thanaraj (2017).

68 McConnell (2022b).

69 Dunn (2017, p. 229).

70 McConnell (2022b).

71 https://www.aspiringsolicitors.co.uk.

72 For example The NRG Group.

73 Rigg (2013).

74 LETR (2013, recommendation 12 p. 289).

75 See also Francis (2015, p. 196) on the importance of institutional reputation to some firms.

76 Boden and Nedeva (2010, pp. 48–49).

77 Bennett et al. (2017).

78 https://www.careers.ox.ac.uk/develop-your-employability-skills#/.

79 Francis (2015, pp. 184-5).

80 LawCareers.Net commercial awareness hub at https://www.lawcareers.net/commercial-awareness and The Lawyer/Lawyer2Be https://www.thelawyer.com/commercial-awareness.

81 Mitchard (2022).

82 Under the SQE students no longer need a qualifying law degree to become a solicitor – the requirement remains for students who want to become a barrister.

83 For example, the package at Clifford Chance https://careers.cliffordchance.com/london/what-we-offer/training-contract.html. First year law students can apply.

84 Rolfe and Anderson (2003).

85 Rolfe and Anderson (2003) and Childs et al. (2014).

86 Holmes (2013).

87 The Teaching Excellence Framework (2022).

88 Ashley and Empson (2013) and Rolfe and Anderson (2003). For recent SRA data on social mobility within law firms see https://www.sra.org.uk/sra/equality-diversity/diversity-profession/diverse-legal-profession.

89 The Purpose Coalition (2021). See also Rare Recruitment https://contextualrecruitment.co.uk/ for law firms using contextualised recruitment practices).

90 For example, in the author’s institution, students participate in CLE during their third year of study.

91 Cotronei-Baird (2020).

92 Dagilyte and Coe (2014).

93 Nicholson (2021).
